# Closed reduction of dorsally displaced distal radius fractures in the elderly provided improved final radiographic results

**DOI:** 10.1186/s13018-023-03733-5

**Published:** 2023-03-27

**Authors:** Sondre Stafsnes Hassellund, Ingrid Oftebro, John Haakon Williksen, Endre Søreide, Jan Erik Madsen, Frede Frihagen

**Affiliations:** 1 Division of Orthopaedic Surgery, Oslo University Hospital, Oslo, Norway; 2 Institute of Clinical Medicine, University of Oslo, Oslo, Norway; 3 Department of Orthopaedic Surgery, Østfold Hospital Trust, Grålum, Norway

**Keywords:** Distal radius fracture, Elderly, Treatment, Non-operative, Closed reduction, Malunion

## Abstract

**Background:**

Recent guidelines recommend non-operative treatment as primary treatment in elderly patients with displaced distal radius fractures. Most of these fractures are closely reduced. We aimed to evaluate the radiological results of closed reduction and casting of dorsally displaced distal radius fractures in patients 65 years or older.

**Methods:**

A total of 290 patients treated during the years 2015, 2018 and 2019 in an urban outpatient fracture clinic with complete follow-up at least 5 weeks post-reduction were available for analysis. Closed fracture reduction was performed by manual traction under hematoma block. A circular plaster of Paris cast was used. Radiographs pre- and post-reduction and at final follow-up were analyzed.

**Results:**

Mean age was 77 years (SD 8) and 258 (89%) were women. Dorsal tilt improved from mean 111° (range 83–139) to 89° (71–116) post-reduction and fell back to mean 98° (range 64–131) at final follow-up. Ulnar variance was 2 mm ((-1)-12) pre-reduction, 0 mm ((-3)-5) post-reduction and ended at mean 2 mm (0–8). Radial inclination went from 17° ((-6)-30) to 23° (SD 7–33), and then back to 18° (0–32) at final follow-up. 41 (14%) patients had worse alignment at final follow-up compared to pre-reduction. 48 (17%) obtained a position similar to the starting point, while 201 (69%) improved. Fractures with the volar cortex aligned after reduction retained 0.4 mm (95% Confidence Interval (CI) 0.1 to 0.7; *p* = 0,022) more radius length during immobilization. In a regression analysis, less ulnar variance in initial radiographs (OR 1.8 (95% CI 1.4 to 2.3) per mm, *p* < 0.001) and lower age (OR 1.06 (95% CI 1.02 to 1.09) per year, *p* < 0.003) protected against loss of reduction.

**Conclusion:**

Subsequent loss of reduction after initial closed reduction was seen in most distal radius fractures. Reduction improved overall alignment in 2/3 of the patients at final follow-up. An aligned volar cortex seemed to protect partially against loss of radial length.

## Background

The treatment of displaced distal radius fractures is under debate, and several authors have shown that malunion after distal radius fractures increases the risk of pain and disability in an adult population [1–3]. Recent reviews and guidelines, however, tend to recommend non-operative treatment for most distal radius fractures in the elderly population [4–6], as patients aged ≥ 65 seem to tolerate fracture displacement and often gain good results without surgery [7–9]. Recent cohort studies and randomized trials comparing surgery and cast immobilization in patients ≥ 65 with dorsally angulated, low energy distal radius fractures conclude that the clinical results are comparable after one year [9–14]. Furthermore, surgery for distal radius fracture in this age group was not found to be cost effective [15].

With this as background, the practical implementation of the non-operative treatment comes into interest. Previous studies on closed reduction have evaluated reduction techniques and prognostic factors for redisplacement [16]. Søsborg-Würtz et al. [17] [18], and radiological parameters such as dorsal displacement, initial ulnar variance, cortical comminution loss of radial inclination and intraarticular involvement have been identified [18–25].

The aim of this study was to assess redisplacement rates and final alignment after closed reduction of unstable distal radius fractures in a population aged ≥ 65 years treated non-operatively. We also aimed to determine radiological factors predicting re-displacement and finally to evaluate complication rates and need for secondary surgeries in patients treated in our unit.

## Patients and methods

We retrospectively evaluated patients ≥ 65 years with a dorsally displaced AO/OTA 2R3 A or C type fracture [26] treated in an urban outpatient fracture clinic. The medical records system identified all patients treated for a distal radius fracture during the years 2015, 2018 and 2019 based on ICD 10-coding. (The gap in the inclusion period was due to inclusion in an RCT.) Patients were included if an initial closed reduction after a displaced distal radius fracture was performed, and radiographs both pre- and post-reduction, and at least 5 weeks post-injury were available. Fractures with initial volar angulation fractures were excluded.

### Treatment

The study was performed in a high-volume centre with experienced cast technicians available. Reduction was done under hematoma block anesthesia. The manual traction technique was used [17]. A circular Plaster of Paris below elbow cast was applied. All patients were scheduled for follow-up in the outpatient clinic, the day after reduction to assess cast tightness and then after 1 and 2 weeks with radiographs to assess fracture alignment. All fractures were immobilized in a cast for 5 weeks, when final radiographs were obtained. Later follow-ups were as clinically indicated.

### Outcome measures

Fractures were classified according to the AO/OTA classification [26]. Standard posterior-anterior (PA) and lateral view radiographs (angled 15 degrees to optimize radiocarpal joint visualization) were evaluated for dorsal tilt, radial inclination, ulnar variance, and intraarticular step off before and after reduction, and after at least 5 weeks. A certified hand surgeon (SH) or an experienced resident (IO) analyzed the radiographs. Prior to the radiological evaluation, the assessors were trained analyzing 30 randomly selected sets of radiographs. An interrater reliability analysis for the AO-classification was performed using Kappa-statistics.[27] We found substantial agreement, free marginal kappa 0.80 (95%CI 0.58–1.00) for the main division of AO/OTA A versus C fractures, and moderate agreement (0.54 (CI 0.32–0.72)) for subgroup classification. Disagreements were discussed in the study group to obtain consensus between the assessors.

Ulnar variance was measured based on the central reference point as described by Medoff [28]. We registered if the volar cortices of the proximal and distal fragments were aligned and in continuity after the initial closed reduction. We also defined “acceptable alignment” based on recommendations for radiological cut-off values for operative management of distal radius fractures in adults below 65 years[5]: (1) dorsal tilt > 100 degrees, (2) ulnar shortening > 3 mm or (3) intra articular step off > 2 mm. In addition we used radial inclination < 15 degrees [29, 30]. The fractures without acceptable alignment according to these criteria at final follow-up were identified as malunions. It was department policy to reduce a fracture even if the displacement was less than the given thresholds [31]. We also categorized the final radiographs in three groups: “worse”, “similar” and “improved” compared to pre-reduction.

From the medical records, we registered any complications and later surgeries related to the fracture such as carpal tunnel release, corrective osteotomies, and extensor pollicis longus (EPL) reconstructions. Data were collected from June 2021 to October 2021. Mean time from injury to the study evaluation was 45 months (range 21–77).

**Ethics**: The study was approved by the local Data Protecting Officer, including waiver for patient consent (20/07430). The study was also reviewed by the Regional Committee for Medical Research Ethics South East Norway and considered not to need approval, ID: 116,564. The patients received a study number and data were maintained unidentifiable throughout the study.

**Statistics:** Statistical analyses were performed using SPSS 26 (IBM Corp, USA) and Excel 16 for Mac (Microsoft Corporation, USA). T-tests were used to compare normally distributed, continuous data, while Pearson’s Chi-square test was used to compare categorical data. Logistic regression was used to evaluate factors predicting redisplacement.

## Results

**Fig. 1:**
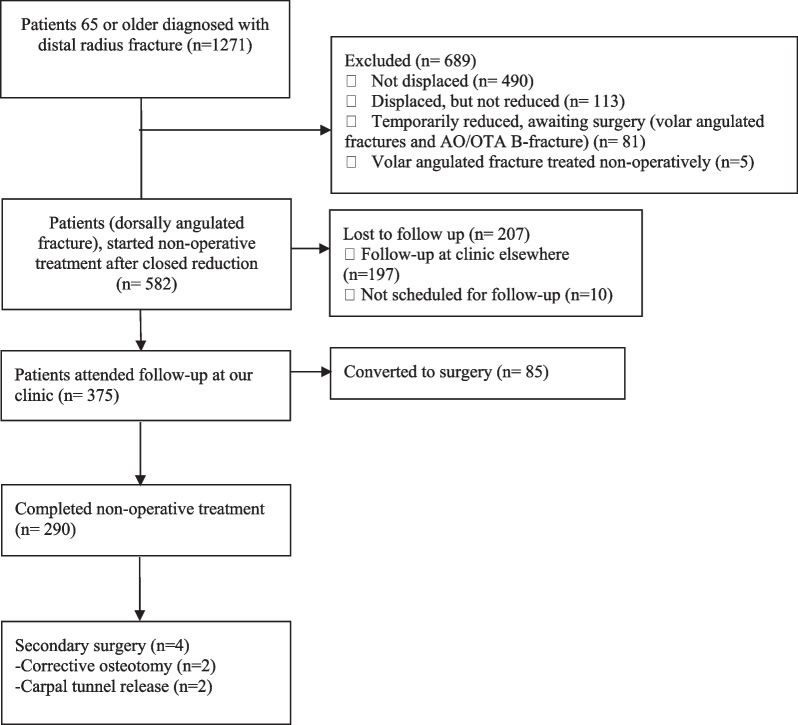
Flowchart Patients diagnosed with distal radius fracture in the inclusion period

We identified 1271 patients aged 65 years or more with a distal radius fracture during the inclusion period (Fig. 1), 582 (46%) patients were treated with initially closed reduction and started non-operative treatment. 290 patients with dorsally displaced fractures matched the inclusion criteria, completed non-operative treatment, and had a complete set of radiographs available for analysis (Table 1). Mean age was 76 years (SD 8) for women and 73 (SD 7) for men.

**Table 1:** Baseline characteristics for the non-operatively treated patients (*n* = 290) A, AO/OTA classification 2R3A; C, AO/OTA classification 2R3C

Patient age; mean (SD)	77 (8)
Women, *n* (%)	258 (89)
Fracture classification	
A2, *n* (%)	17 (6)
A3 *n* (%)	142 (49)
C1 *n* (%)	27 (9)
C2 *n* (%)	51 (18)
C3 *n* (%)	53 (18)

**Fig. 2:**
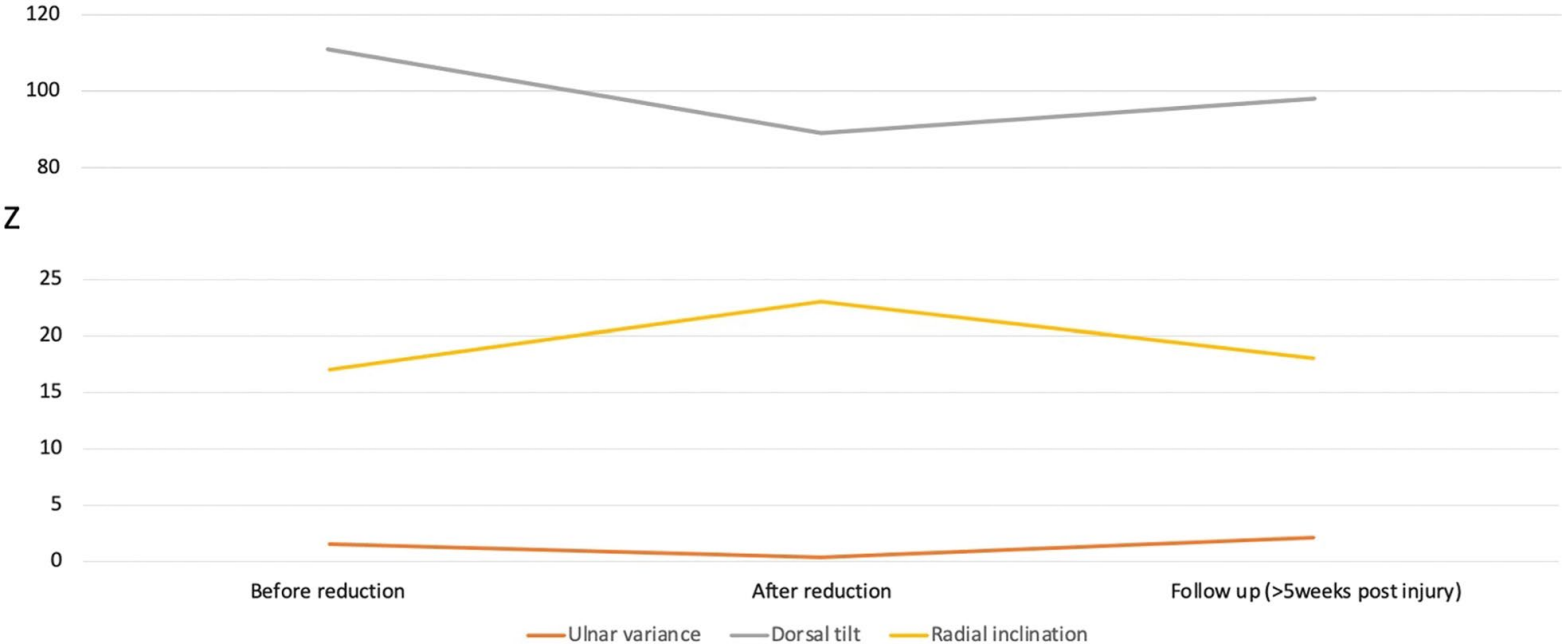
Mean value of the radiological parameters before and after reduction and at final follow-up. Ulnar variance measured in mm, dorsal tilt, and radial inclination in degrees. See Table 2 for statistics

**Fig. 3:**
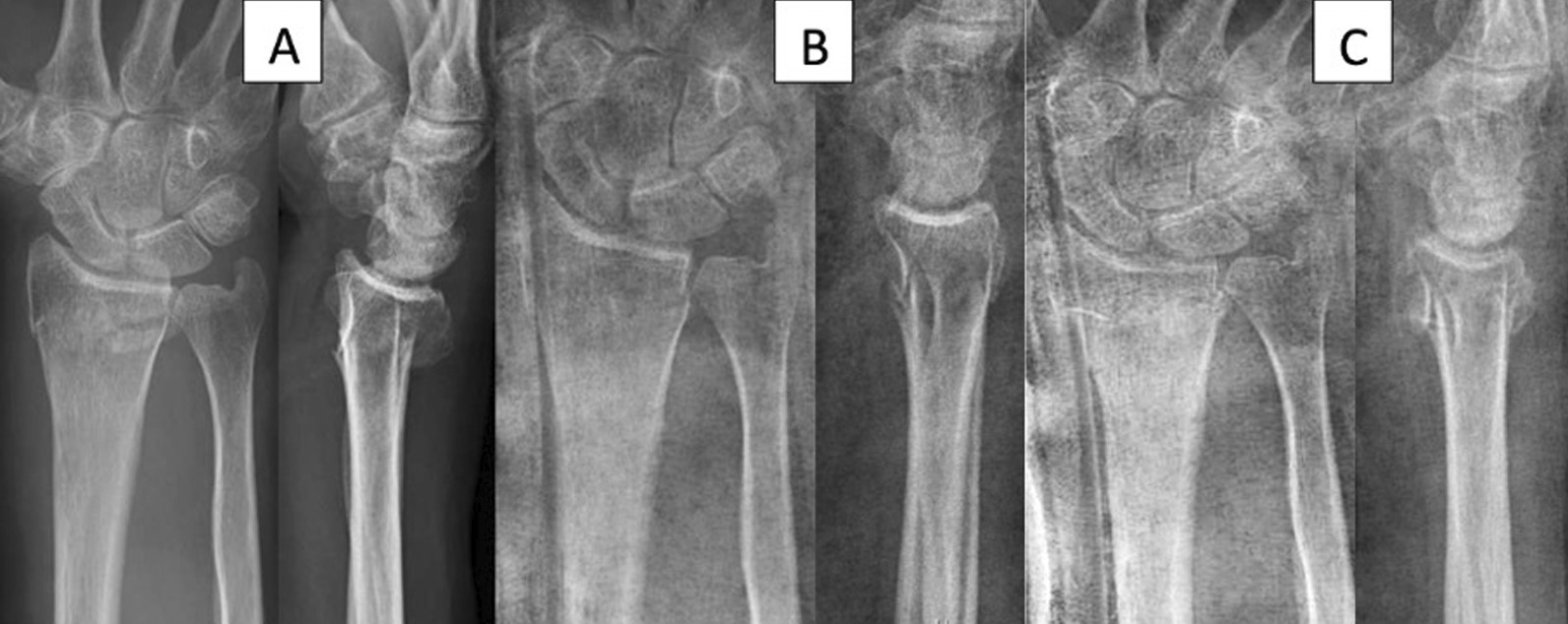
Example radiographs. 73 yo female, fall from own height on extended wrist. Pre (**A**) and post (**B**) reduction, and at follow-up (six weeks, **C**). Compared to pre-reduction, dorsal tilt has improved, but radius has become slightly shorter at follow-up. Reprinted with patient´s permission. Department of Radiology, Oslo University Hospital

Of the 290 included fractures, 268 (92%) showed dorsal comminution. All but one fracture improved alignment radiologically after the initial closed reduction, while all but three patients had some loss of reduction from post-reduction radiographs to the final follow-up (Table 2, Fig. 2). Mean deterioration during the immobilization period was 9 degrees dorsal tilt, 5 degrees radial inclination and 2 mm ulnar variance (Fig. 3). At final follow, mean ulnar variance was higher than pre-reduction, but the other radiographical parameters improved. (Table 2, Fig. 2). 58 (20%) patients had an increased dorsal tilt at FU compared to the initial radiographs before reduction, but less than 5 degrees in 29 of them. Overall, we found radiological fracture alignment to be improved in 201 (69%) comparing pre-reduction radiographs to final follow-up. 48 (17%) had a similar radiological position as pre-reduction, while 41 (14%) patients had a worse radiological position at final follow-up.

**Table 2:** Radiological results after non-operative treatment (*n* = 290) pre- and post-reduction and at final follow-up

	Pre-reduction	Post-reduction	Final follow-up	Mean difference pre-reduction and final follow-up (95% CI)	*p*-value
Dorsal tilt (degrees, range)	111 (83–139)	89 (71–116)	98 (64–131)	13 (11.2–14.7)	< 0.001
Radial inclination (degrees, range)	17 ((-6)-30)	23 (7–33)	18 (0–32)	− 1 (( − 1.7)) to ( − 0.3))	0.003
Ulnar variance (mm, range)	2 (1–12)	0 ((-3)-5)	2 (0–8)	-0.5 ((-0.7)-0.4)	< 0.001
Intraarticular step > 2 mm *n* (%)	15 (5)	2 (1)	9 (3)		< 0.001*
Acceptable alignment; *n* (%)	28 (10%)	257 (89%)	118 (41%)		< 0.001*

Mean difference and *p*-value is based on the difference between pre-reduction and final radiographs. CI: Confidence interval

*p*-value from paired samples t-test. *p-value from Pearson Chi square

118 (40%) of the treated fractures were categorised as “volar cortex aligned” after reduction. The degree of redisplacement based on whether the volar cortex was aligned after reduction or not, was not statistically different for dorsal tilt or radial inclination, but they retained radial length better, as there was less increase in ulnar variance. (Table 3).

**Table 3:** Radiological results based on whether volar cortex was aligned or not after reduction

	Volar cortex not aligned (*n* = 117)	Volar cortex aligned (*n* = 173)	Mean difference (95%CI)	*p*-value (Independent sample t-test)
*Dorsal tilt (degrees)*				
Pre-reduction (range)	112 (83–139)	110 (83–134)	2 (0–5)	0.091
Post-reduction (range)	90 (71–116)	87 (75–101)	3 (2–5)	< 0.001
Final follow-up (range)	98 (68–131)	97 (64–123)	1 (( − 2)-4)	0.467
*Radial inclination (degrees)*				
Pre-reduction (range)	15 (( − 3 to 29)	18 (6)	− 3.0 ( − 4.6 to ( − 1.3))	< 0.001
Post-reduction (range)	22 (7–30)	24 (4)	− 2.0 ( − 3.1 to ( − 1.0))	< 0.001
Final follow-up (range)	16 (0–31)	20 (5)	− 3.4 ( − 4.9 to ( − 1.9))	< 0.001
*Ulnar variance (mm)*				
Pre-reduction (range)	2 (0–7)	1 ((− 1)-9)	0.6 (0.2–1.0)	0.007
Post-reduction (range)	0 (( − 3 to 4))	0 ((− 3)-5)	0.3 ( − 0.0 to 0.5)	0.069
Final follow-up (range)	3 (0–8)	2 (0–7)	1.0 (0.5–1.4)	< 0.001
*Difference primary radiographs to final follow-up*				
Dorsal tilt (SD)	14 (15)	12 (14)	1.2 ( − 2.5 to 5.0)	0.513
Radial inclination (SD)	1 (6)	1.3 (6)	− 0.5 ( − 1.9 to 1.0)	0.530
Ulnar variance (SD)	1 (1.5)	0.3 (1.3)	0.4 (0.05–0.75)	**0.022**

In a logistic regression analysis, ulnar variance pre-reduction (*p* =  < 0.001) and age (*p* = 0.002) were significant predictors for malunion (Table 4) Ulnar variance was the most important factor; 59 (89%) of the 66 fractures (22%) with 2 mm or more ulnar variance pre-reduction were malunited at final follow-up. Statistical significant values in bold.

**Table 4:** Correlation between final malunion and patient factors and primary radiographs

Parameter	Bivariate analysis, *p*-value	Logistic regression, *p*-value	Odds ratio (95% CI)
Age	** < 0.001**	**0.003**	1.06 (1.02 to 1.09)
Ulnar variance	** < 0.001**	** < 0.001**	1.84 (1.44 to 2.36)
Sagittal tilt	**0.004**	0.770	1.00 (0.98 to 1.03)
Step > 2 mm	0.236	0.452	1.65 (0.45 to 6.05)
Radial inclination	** < 0.001**	0.705	0.99 (0.95 to 1.04)
Dorsal comminution	0.963	0.092	0.44 (0.17 to 1.14)

Four of 290 (1%) patients later underwent secondary surgery related to their distal radius fracture. Two patients needed a corrective osteotomy, and two patients had surgery for carpal tunnel syndrome. In addition, two patients were treated for complex regional pain syndrome (CRPS). Statistical significant values in bold. 

## Discussion

Our results confirm that most displaced distal radius fractures treated non-operatively in elderly patients redisplace to some extent after initial closed fracture reduction. Even so, 41% of the fractures with unacceptable alignment before closed reduction had maintained acceptable alignment at the final follow-up. About one out of seven fractures had a worse radiological position at final follow-up compared to pre-reduction images. The main predictors for redisplacement in the present patient series were initial shortening of the radius and high age. Furthermore, lack of volar cortex alignment resulted in increased loss of radial length during the immobilization period.

Assessment of instability of distal radius fractures has been extensively studied. Lafontaine et al. [19] identified five factors that indicated instability and secondary displacement; dorsal comminution, dorsal angulation > 20 degrees, intraarticular fracture, associated fracture of the ulna and age > 60. They postulated that fractures exhibiting more than three of these instability criteria needed extra radiological surveillance due to the increased risk of redisplacement. Nesbitt et al. [20] later included 50 patients with an age of 60 based on Lafontaine´s 5 instability criteria. They found that 46% maintained adequate reduction throughout the non-operative treatment, compared to our rate of 41%.

Hove et al. [25] analyzed 645 non-operatively treated distal radius fractures in patients with a mean age of 61 years. They observed an average radial shortening throughout the cast immobilization period of 3 mm, as compared to our 1.7 mm, and an increase in dorsal angulation of 7 degrees compared to our 9 degrees. Their conclusion also compared well with our findings, in that initial shortening of the radius was the main predictor for a malunion. In a larger study evaluating approximately 4000 distal radius fractures in patients aged mean 59 years, Mackenny and co-authors[21] found age, initial ulnar variance, and initial dorsal comminution to be the main predictors for fracture displacement and malunion.

Several authors have found that dorsal fracture comminution increase the risk of malunion [22, 23], Wadsten and collaborators [23] found that both volar and dorsal comminution predicted later displacement in a prospective study including 389 fractures. In Makhni et al.' study [22] fractures with dorsal comminution (62% of the fractures) had a displacement rate of 75% compared to a rate of 45% in the fractures without comminution. In our study, we could not identify dorsal comminution as an independent predictor for redisplacement, but only 22 (8%) of our patients did not exhibit dorsal fracture comminution.

Even though most of these mentioned studies were performed in mixed-age populations, their findings compare well with ours in an elderly population. It seems that the redisplacement risk continues to increase with age, even beyond the age of 65. Few authors have, however, addressed the specific effects of the initial closed fracture reduction in the non-operative treatment of distal radius fractures in the elderly. Beumer et al. [32] included only low demand or demented elderly with a mean age of 82. They found that only 7 of 44 (16%) dorsally displaced fractures had acceptable alignment after 6 weeks, and based on this finding, they considered the importance of fracture reduction in elderly, frail patients to be questionable. Our results in a larger and more heterogenous group of elderly patients demonstrate a lower redisplacement rate after closed reduction.

The present study has inherent weakness. Even though the sample size is large, 197 (38%) patients were followed up elsewhere, and, consequently, the number of surgeries after fracture healing might be underestimated. Also, we do not know is the fracture distribution in the study is representative for the whole fracture-population. Furthermore, we were not able to correlate our radiographic findings to functional outcomes. Some patients did not have available radiographs later than five weeks after injury. Therefore, further displacement might have occurred. However, recent publications have reported minimal radiological deterioration after 6 weeks in patients over 65 years and over 50 years [9, 33]. Also, the judgement of whether final alignment was better or worse than pre-reduction was difficult for some cases, and hence, some subjective judgement was required. Also, radiological measurements are unprecise and vary between observers. However, the observed changes between pre-reduction, post-reduction and final radiographs were similar between the observers. The study was performed in a high-volume centre. The results of the closed manipulation may be less favourable in a low volume setting. Even so, we believe that the study provides interesting information on the radiological effects of initial fracture reduction and later redisplacement during non-operative treatment in an elderly population.

## Conclusion

Initial fracture reduction does not prevent redisplacement, but our study demonstrates the benefit of initial closed fracture reduction with improved the radiological alignment of displaced distal radius fractures in 2 of 3 patients at final follow-up in this study group. Closed reduction is a simple and easily available method to improve alignment, most notably dorsal tilt. Since we do not know the natural history of un-reduced displaced distal radius fractures in the elderly, we therefore continue to recommend initial closed reduction in most displaced distal radius fractures in elderly patients.

## Data Availability

Data files with radiological parameters are available.
